# Effect of a generative artificial intelligence digital scribe on pediatric provider documentation time, cognitive burden, and burnout

**DOI:** 10.1093/jamiaopen/ooaf068

**Published:** 2025-07-03

**Authors:** Jonathan H Pelletier, Kevin Watson, Jenny Michel, Robert McGregor, Sarah Z Rush

**Affiliations:** Department of Clinical Informatics, Akron Children’s Hospital, Akron, OH, 44308, United States; Division of Pediatric Critical Care Medicine, Department of Pediatrics, Akron Children’s Hospital, Akron, OH, 44308, United States; Department of Clinical Informatics, Akron Children’s Hospital, Akron, OH, 44308, United States; Division of Pediatric Gastroenterology, Department of Pediatrics, Akron Children’s Hospital, Akron, OH, 44308, United States; Division of Pediatric Emergency Medicine, Department of Pediatrics, Akron Children’s Hospital, Akron, OH, 44308, United States; Department of Pediatrics, Akron Children’s Hospital, Akron, OH, United States; Department of Clinical Informatics, Akron Children’s Hospital, Akron, OH, 44308, United States; Division of Pediatric Hematology/Oncology, Department of Pediatrics, Akron Children’s Hospital, Akron, OH, 44308, United States

**Keywords:** artificial intelligence, documentation, burnout, pediatrics

## Abstract

**Objective:**

To assess the effect of a digital scribe among pediatric providers on documentation time, cognitive load, burnout, and caregiver satisfaction.

**Materials and Methods:**

Six-month pilot of digital scribe use in pediatric generalists and subspecialists between June 27, 2024 and February 22, 2025. We included all pilot providers with EHR record logs. Using pretest-postest and interrupted time series analysis (ITSA), we analyzed associations between digital scribe use and documentation time, NASA task load index, mini-Z burnout inventory, and caregiver satisfaction. We conducted thematic analysis of provider feedback.

**Results:**

The digital scribe was used to generate notes for 69.5% of encounters (31 931/45 914) across 84 providers during 1454 provider-weeks. Compared with before scribe adoption, there was a mean time savings of 2.8 (95% CI, 2.1-3.5) minutes in the EHR notes activity per appointment following digital scribe adoption, averaging 1.5 (95% CI, 1.1-1.8) hours per provider-week, totaling 2143 (95% CI, 1607-2678) hours during the pilot. ITSA demonstrated 20.9% (95% CI, 17.2-24.6) time savings with digital scribe adoption. In provider surveys, there were significant reductions in all task load components (all *P <* .001). Burnout decreased from 54.9% (28/51) to 33.3% (17/51), *P =* .01. Provider-specific caregiver’s likelihood-to-recommend increased from 92.3% (IQR, 88.1%-95.5%) to 94.3% (IQR, 89.7%-100.0%), *P =* .01. Thematic analysis of provider feedback frequently identified decreased cognitive burden and time savings.

**Discussion:**

Digital scribe use was associated with decreased documentation time, task load, provider burnout, and improved caregiver satisfaction. Further study is needed to determine if pilot results are scalable.

## Introduction

Clinicians’ notes serve myriad functions.^1^ In addition to facilitating care, documentation is used for reimbursement, quality improvement, research, safety reporting, regulatory audits, and malpractice defense.^2–4^ Implementation of electronic health records (EHRs) was coupled with pressure to lengthen documentation to support these functions.^5^^,^^6^ Together, electronic data entry, regulatory requirements, and social pressures resulted in a higher burden on clinicians.^7^ Electronic tools such as “copy and paste” have further lengthened notes without reducing clinician time.^8–10^ The overall effect has been increased documentation time compared to paper records, resulting in less face-to-face time between providers and patients.^11–13^ The problem is most notable in the United States, where documentation is 4-times longer and 25% more time-consuming than peer countries.^5^^,^^14^

Documentation burden is one important factor in clinician burnout.^15–23^ Approximately half of providers report burnout, though results are heterogenous by specialty.^24–27^ Clinicians suffering from burnout consistently report EHR frustrations as a contributing factor.^28–31^ Increased EHR usage time is associated with burnout, particularly charting outside of scheduled work hours.^15–23^ However, burnout appears to be related both to task duration and complexity. Improving EHR usability reduces provider cognitive load, which improves clinical performance and reduces burnout.^32–40^ Thus, documentation burden should be considered both in terms of time and cognitive strain.

One method of reducing documentation burden is by using medical scribes. Human scribes have been associated with decreased provider EHR time and clinician burnout.^41–44^ However, human scribes typically cost tens of thousands of dollars per year, which must be offset by increasing patient encounters.^45^^,^^46^ Additionally, human scribes require dozens of hours of training and still may not achieve competency.^47^ Human scribes may also suffer burnout themselves.^44^

Digital scribes are a promising alternative to human scribes.^48–50^ Digital scribes receive recordings of clinical conversations and use automatic speech recognition (ASR) algorithms to generate transcriptions, which are subsequently used as input to generative artificial intelligence models to create a structured clinical note.^48^^,^^51–54^ Digital scribes are associated with decreased EHR time and improved workflow satisfaction.^55–58^ However, existing studies are limited to adult providers.^55–58^ While EHR documentation time is similar between adult and pediatric providers, time savings from digital scribe use may vary in pediatrics due to challenges in simultaneously obtaining history from patients and caregivers.^59^ Furthermore, existing studies have not systematically assessed provider cognitive burden when using a digital scribe and have come to inconsistent conclusions regarding provider burnout.^55–58^ Prior work at our institution had noted reports of provider burnout consistent with national averages. Prior informatics projects (eg, redesigning note templates to reduce EHR time) had not shown significant improvement in provider burnout. Given that our institutional experience appeared to mirror US national healthcare trends, we sought to analyze the effect of implementing a digital scribe in a heterogenous sample of pediatric healthcare providers.^60^

## Methods

### Study population

This protocol was approved by the Akron Children’s Hospital Institutional Review Board. In 2025 Akron Children’s Hospital underwent a staged pilot trial of a commercial digital scribe (Abridge, Philadelphia, PA) to generate clinical notes and patient instructions from ambulatory pediatric encounters.^60^ A business associate agreement was signed between the health system and the digital scribe vendor, and a cybersecurity review was completed as per institutional policy. As the goal of the program was to reduce clinician EHR time and burnout, the decision to undertake a staged pilot was motivated by a desire to constrain program costs while not requiring funding from individual physicians or changes in clinical productivity expectations.^61^ The choice of digital scribe vendor was determined by independent rankings of commercially available digital scribe products.^62^ This study included clinicians who completed a pilot trial of the digital scribe software program between June 27, 2024 and February 22, 2025. Clinical psychologists were excluded, as they do not have EHR usage records in our system. The health system initially offered digital scribe licenses to all clinicians from 3 high-volume outpatient pediatric subspecialty departments (cardiology, endocrinology, and gastroenterology). Licenses from those who declined to participate were reallocated to other clinicians on a voluntary basis. An initial analysis of digital scribe adoption was performed after 30 days. Because only volunteer status and clinical volume predicted scribe adoption, the organization then serially increased the license count. During the rolling expansion, volunteer recruitment was broadened to multiple other high-volume outpatient offices (allergy and immunology, dermatology, orthopedics, general pediatrics, nephrology, neurology, neurosurgery, psychiatry, and sports medicine).

### Digital scribe workflow

**Figure 1.**
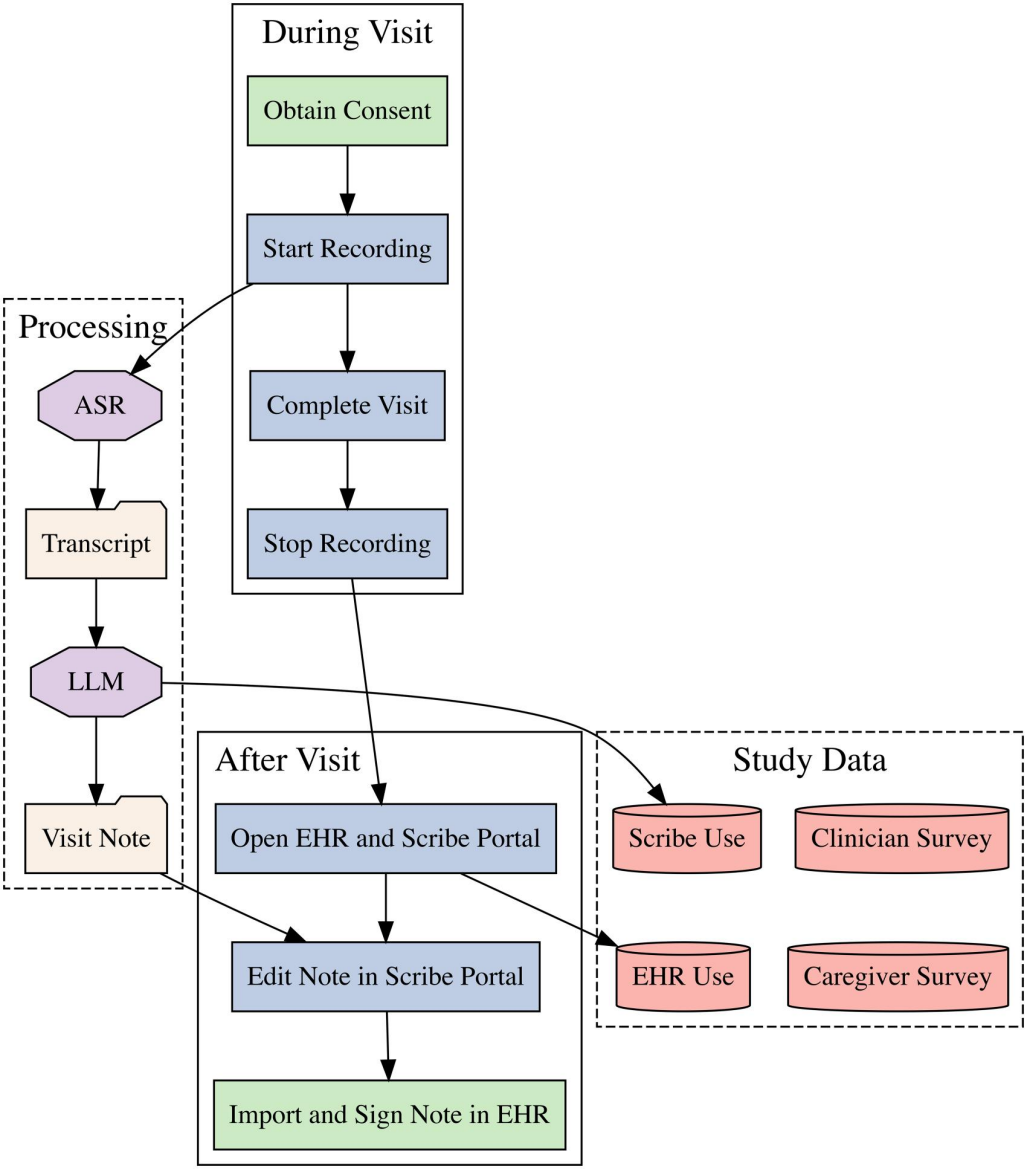
Workflow diagram for digital scribe use. Rectangles represent clinician workflow. Green rectangles represent process start and stop, while blue rectangles represent intermediate steps. Purple octagons represent computations, and manilla files represent computational products. Red cylinders represent data storage for the present study. Abbreviations: ASR: automated speech recognition; EHR: electronic health record; LLM: large language model.

The use of the digital scribe in clinical practice is outlined in Figure 1. Clinicians received a 1-hour virtual training on software use prior to implementation. Caregivers provided verbal consent and patients provided assent prior to audio recording. Following consent, clinicians recorded clinical encounters using personal smartphone devices’ speakerphone capabilities.^52^ HIPAA-compliant encrypted recordings were transmitted to United States-based cloud servers with no local storage on personal devices.^52^ All data were encrypted in transit using Transport Layer Security 1.2 or higher and at rest using Advanced Encryption Standard with a 256-bit key length.^52^ Recordings were transcribed using commercial automated speech recognition (ASR) models trained on a dataset of clinical audio with associated human transcription, clinical notes, and patient metadata.^52^ ASR model derivation and performance has been previously published.^52–54^^,^^60^ Following ASR, clinical notes were generated from transcripts by commercial large language models (LLMs) tuned with proprietary prompt engineering and reinforcement learning by clinical reviewers.^52–54^^,^^60^^,^^63^ Following the patient encounter, clinicians opened the electronic health record (EHR; Epic, Verona, WI) and a web-based portal provided by the digital scribe vendor (Abridge, Philadelphia, PA). Clinicians reviewed and edited the LLM-generated progress note. Clinicians also had the option to review the ASR-generated encounter transcript and to generate a patient summary designed at an 8th-grade reading level. After review, clinicians transferred the progress note from the web-based portal into the EHR via embedded SmartPhrases. The notes were signed by the clinician at the close of the encounter as per the standard workflow. Notes were intermittently audited by health information management and revenue cycle specialists per typical institutional workflow.

### Data sources

Study data were provided from 4 sources. First, data regarding software use, number of notes generated, and provider note feedback were extracted at the encounter-level from the digital scribe vendor. Second, weekly provider-level documentation length and time in notes and monthly provider-level EHR usage logs were extracted from the EHR analytics portal (Epic Signal, Verona, WI). EHR usage logs were queried from November 30, 2023 through February 22, 2025 (the latest date through which data were available) to ensure at least 30 weeks of pre-scribe data for interrupted time series analysis. Third, optional surveys were sent to providers before and 4 weeks after use of the digital scribe. These surveys measured self-reported EHR time, note-specific cognitive burden, burnout, self-assessed visit quality, and job satisfaction. Note-specific cognitive burden was measured using the mental demand, temporal demand, and performance indices of the NASA Task Load Index. The NASA Task Load Index is a measure of cognitive task burden that has been previously validated in multiple healthcare settings.^64–68^ It is the most commonly used index to measure EHR burden; higher scores have been correlated with worse clinical performance and provider burnout.^33^^,^^34^^,^^38^^,^^39^^,^^69^^,^^70^ Burnout was assessed using the burnout index of the Mini-Z survey. The Mini-Z survey has been previously validated against the Maslach Burnout Inventory and is commonly used for healthcare research due to brevity.^20^^,^^71^^,^^72^ Fourth, monthly provider-level caregiver satisfaction data was extracted from post-encounter survey results provided routinely after ambulatory clinical encounters at our institution (NRC Health, Lincoln, NE). These surveys are sent electronically or via mail to patient’s caregivers following a subset of encounters.

### Statistical analysis

Digital scribe use and note generation were described with summary statistics. EHR usage, provider survey responses, and caregiver satisfaction survey data before and after digital scribe implementation (“pre-scribe” and “post-scribe,” respectively) were compared using paired t-tests with grouping at the provider-level. The reporting period during which the software was implemented by each provider was censored to ensure that pre- and post-implementation periods were representative. As before-and-after analysis may be subject to confounding by other similarly timed unknown practice changes, EHR usage data were subsequently normalized at the provider level prior to digital scribe implementation to allow for interrupted time series analysis and determine the immediate effect of digital scribe adoption.^73–76^ Last, because multiple studies have demonstrated the ability of LLMs to expedite analyses of qualitative results,^77–82^ we used a general purpose LLM (GPT-4, OpenAI, San Francisco, CA, United States) to assist in thematic analysis of free-text provider comments. The LLM was used to generate lists and counts of frequently represented themes in provider survey data and requested to generate representative quotes. The LLM was specifically prompted to include both emotionally positive and negative themes.^77–82^ All representative provider comments were human verified to protect against the low rates of hallucinations reported in the literature for these use cases.^77–82^

### Competing interest

The digital scribe vendor had no role in the decision to publish the manuscript. The vendor supplied data for the manuscript as outlined above and was given the opportunity to review the methods for accuracy in description. No financial payment or in-kind support was provided for the study.

## Results

### Initial digital scribe adoption and refusal

There were a total of 65 providers to whom a digital scribe license was offered in the initial 30 days. Of those, 47/65 (72.3%) adopted the digital scribe. At baseline, those who adopted the virtual scribe had a higher proportion of days with clinic appointments (median 46% [IQR, 36%-55%] vs 37% [IQR, 26%-51%], *P =* .049) and a lower proportion of same-day chart closure (median 79% [IQR, 42%-94%] vs 94% [IQR, 64%-98%], *P =* .049) compared to those who declined the virtual scribe. In addition, 92.9% (13/14) volunteers compared with 66.7% (34/51) of randomly assigned providers adopted the virtual scribe (*P =* .052). In multivariable regression, only volunteer status (OR 8.93 [95% CI, 1.42-179]) and percentage of days with clinic appointments (OR 1.06 [95% CI, 1.01-1.11]) remained significant predictors of scribe adoption. Because of this, the institution began recruiting volunteers from any high-volume outpatient department for subsequent rolling pilot expansion.

### Digital scribe use and time savings

**Figure 2.**
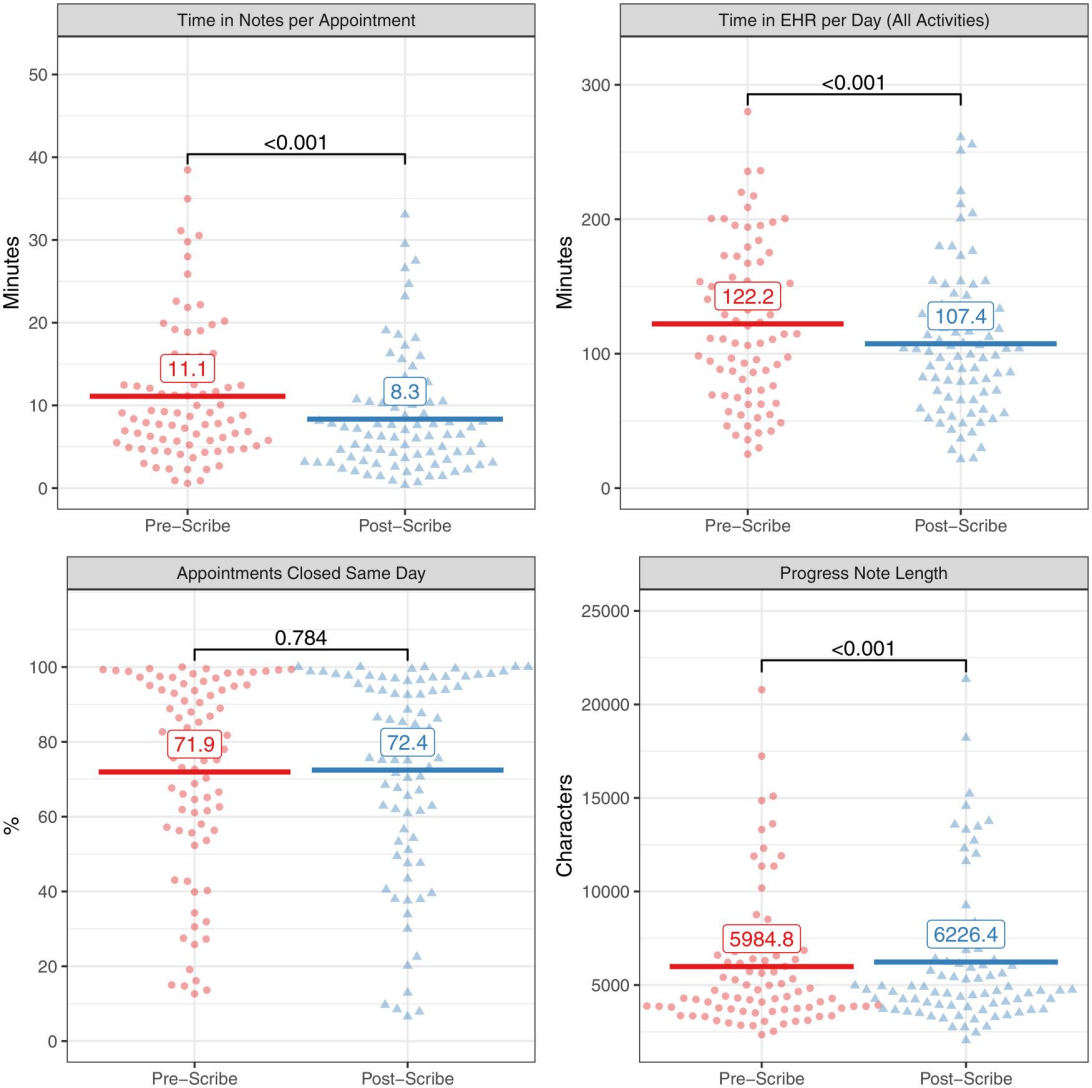
Electronic health record usage logs for digital scribe pilot users. The x-axis shows the time period, and the y-axis shows the metric for each usage log. Each panel has a separate y-axis as shown. The usage log type is displayed in the gray bar above each panel. For each panel, the horizontal lines and numbers represent the mean value for each time period, and the *P*-values between the groups represent paired t-test values. Each individual point represents a single provider per time period. The shapes and colors of the points correspond to the time periods.

**Figure 3.**
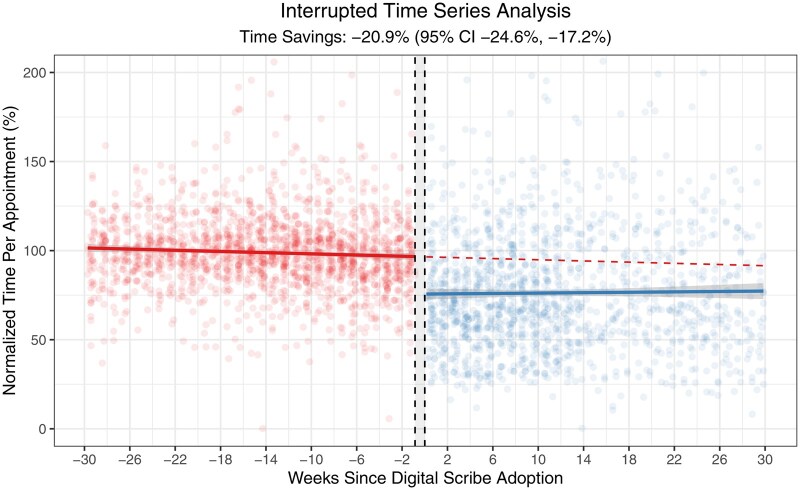
Interrupted time series analysis of time in notes per appointment. The x-axis shows the reporting weekly EHR usage log reporting period start date relative to an individual provider’s adoption of the digital scribe. The y-axis shows the weekly time in notes per appointment normalized to 100% of each provider’s pre-implementation weekly time. Each point represents an individual provider-week. Colors denote points before and after digital scribe implementation. The red line shows a time series model trained on pre-implementation data, and the blue line shows a time series model trained on post-implementation data. The gray-shaded regions represent the 95% confidence interval of each model. The red dashed line shows the time in notes predicted by the pre-implementation model (the time in notes that would have been expected if the digital scribe had not been implemented).

The rolling expansion of the pilot included 87 total providers (47 that adopted the digital scribe during the first 30 days and 40 that adopted the scribe after the first 30 days). There were 3 clinical psychologists who used the digital scribe but were excluded from the analysis due to lack of EHR usage log data. Thus, analysis was performed on 96.6% (84/87) providers who completed the digital scribe pilot. The digital scribe was used to generate notes for 69.5% of encounters (31 931/45 914) across 84 providers between June 27, 2024 and February 22, 2025. Individual providers used the digital scribe for a median of 79.0% of their encounters (IQR, 50.8%-91.2%). EHR logs are shown in Figure 2. There was a mean time savings of 2.8 (95% CI, 2.1-3.5) minutes in the EHR notes activity per appointment following digital scribe adoption. There were 45 914 appointments across 1454 provider-weeks in the pilot period. Based on this mean time savings of 2.8 (95% CI, 2.1-3.5) minutes per appointment, the total time savings across the pilot was 2143 (95% CI, 1607-2678) hours in notes. This represented a savings of 1.5 (95% CI, 1.1-1.8) hours per provider-week. When considering all EHR time (in any activity), there was a mean time savings of 14.8 (95% CI, 10.4-19.1) minutes per day. There were 7277 provider-workdays in the pilot period. Based on this mean time savings of 14.8 (95% CI, 10.4-19.1) minutes per day, the total time savings across the pilot was 1795 (95% CI, 1261-2316) hours in the EHR. This represented a savings of 1.2 (95% CI, 0.9-1.6) hours per provider-week. There was no difference in time outside scheduled hours per day (26.4 [SD 22.6] minutes vs 28.5 [SD 23.2] minutes per day, *P =* .11). Interrupted time series analysis is shown in Figure 3. Digital scribe implementation was associated with an immediate 20.9% (95% CI, 17.2-24.6) reduction in time in notes per appointment (*P <* .001). There was no significant effect of time after digital scribe implementation, though time in notes decreased by 0.2% (95% CI, 0.0-0.3) in the 30 weeks prior to scribe implementation (*P =* .049).

### Provider and patient surveys

**Figure 4.**
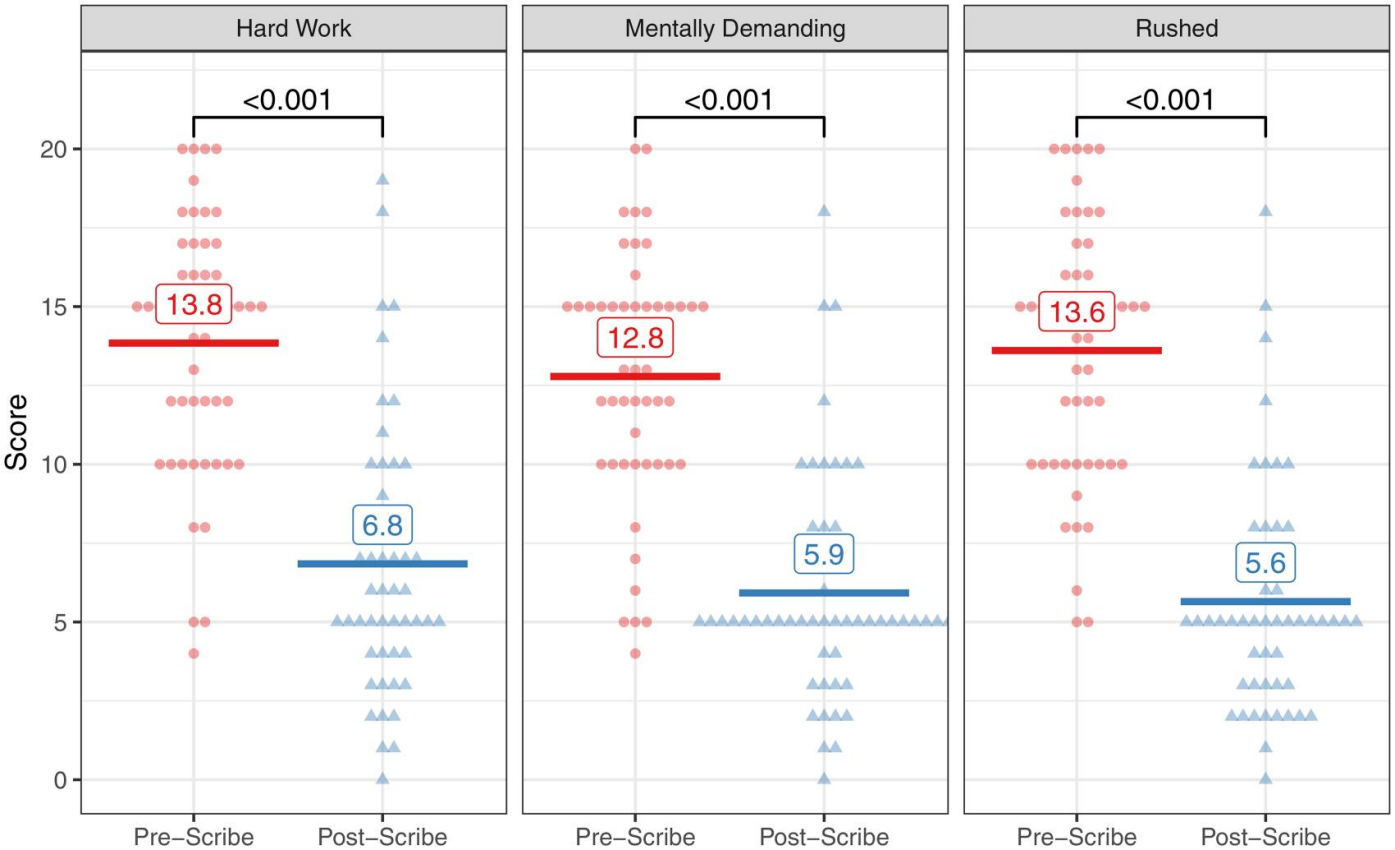
NASA task load index survey results for digital scribe pilot users. The x-axis shows the time period, and the y-axis shows the score. All panels use a 0-20 score. For each panel, the horizontal lines and numbers represent the mean value for each time period, and the *P*-values between the groups represent paired t-test values. Each individual point represents a single provider per time period. The shapes and colors of the points correspond to the time periods.

**Figure 5.**
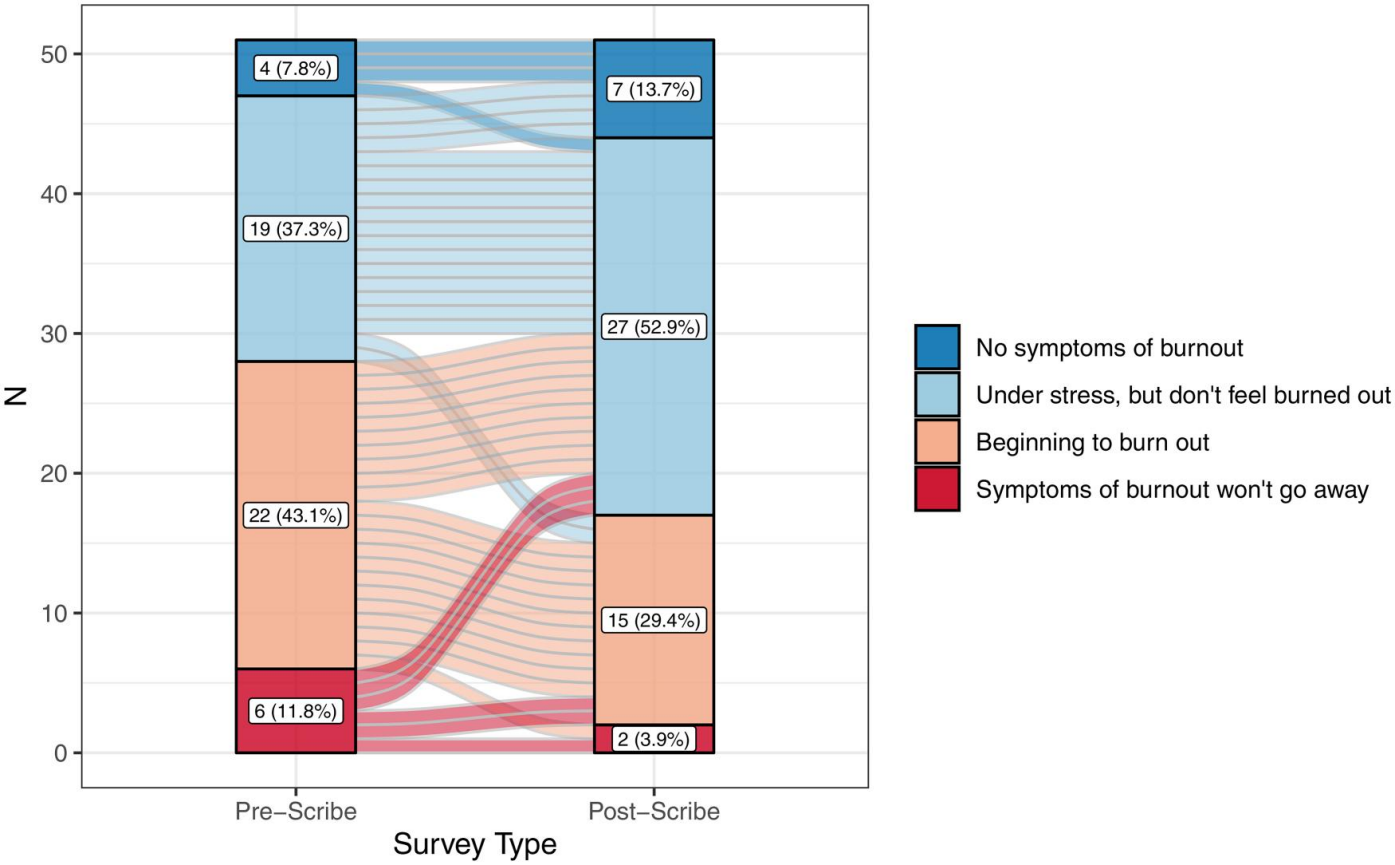
Alluvial plot of burnout survey results for digital scribe pilot users. The *x*-axis shows the time period, and the *y*-axis shows the number of respondents in each category. The color-shaded regions represent the number of respondents in each category, shown by the color scale on the right. The numbers within the bars show the number and percent of respondents in each category. Each horizonal line represents the serial answers for a given survey respondent.

Both pre- and post-scribe surveys were completed by 60.7% (51/84) providers. After digital scribe use, there was a decrease of 1.1 (95% CI, 0.4-1.8) in self-reported hours writing notes outside work per week (2.8 [SD 2.3] hours vs 3.9 [SD 2.7] hours, *P =* .004). Results of the provider NASA Task Load Index surveys are shown in Figure 4. On a 0-20 scale, there was a reduction of 6.9 points (95% CI, 5.7-8.1) in providers’ scoring of the mental demand of note writing. There was an 8.0 (95% CI, 6.6-9.3) point reduction in providers’ scoring of how rushed they felt during note writing, and a 7.0 (95% CI, 5.5-8.5) point reduction in providers’ scoring of how hard providers felt they had to work to accomplish their level of note-writing performance. Results of the provider burnout survey are shown in Figure 5. When responses were dichotomized, 33.3% (17/51) providers reported feeling burned out on the post-scribe survey compared to 54.9% (28/51) in the pre-scribe survey [*P =* .01]. In addition, 76.5% (39/51) providers agreed or strongly agreed that “[the digital scribe] has increased my satisfaction at work.” The proportion of providers who agreed or strongly agreed that they could “give my patients my undivided attention” increased after digital scribe adoption (94.1% [48/51] vs 56.9% [29/51], *P <* .001); 56.9% (29/51) providers agreed or strongly agreed that “[the digital scribe] has improved the quality of my notes.” However, the proportion of providers who agreed or strongly agreed that patients could understand their care plan did not significantly change (74.5% [38/51] vs 66.7% [34/51], *P =* .48). The proportion of providers who agreed or strongly agreed that they could add another patient to their daily schedule did not significantly change (66.7% [34/51] vs 70.6% [36/51], *P =* .80).

Caregiver survey results were available for 96.4% (81/84) of providers. The median provider-specific likelihood to recommend increased after digital scribe implementation to 94.3% (IQR, 89.7%-100.0%) compared with 92.3% (IQR, 88.1%-95.5%) before digital scribe use (*P =* .01). There was no significant change in the median provider-specific response to “my provider listened” after digital scribe implementation (87.9% [IQR, 80.0%-95.8%] vs 86.5% [IQR, 82.6%-89.7%], *P =* .36). There was no significant change in the median provider-specific response to “I trust my provider with my care” after digital scribe implementation (88.5% [IQR, 82.9%-95.0%] vs 87.9% [IQR, 83.3%-90.7%], *P =* .26).

### Provider feedback

Providers submitted 47 unstructured comments on the use of the digital scribe. Overall 83.0% (39/47) comments were positive, and 17.0% (8/47) were negative. Thematic analysis of provider comments is shown in Table 1. There were a total of 7 themes identified with 61 total thematic occurrences (some comments addressed multiple themes). The most frequently identified themes were that the digital scribe allowed for reduced documentation cognitive burden, decreased documentation time, and improved work-life balance. Negative themes related to concerns related to overly general summarizations, lack of customization in behavioral health notes, and patient privacy and consent logistics.

**Table 1.** Thematic analysis of provider comments.

Theme, occurrence, and description	Representative quote
**Reduced documentation burden (13 occurrences)**—Providers frequently reported that the scribe significantly reduced the cognitive and time burden associated with notetaking, making clinical documentation faster and less stressful.	“*[The scribe] has lifted a layer of dread that I felt when faced with each patient encounter. It allows me to enjoy what I do.*” “*I'm not worried that I am going to forget some important detail. If I get pulled away by some emergent OR case, I don't have the burden of documentation hanging over my head.*”
**Increased efficiency and speed (11 occurrences)**—Many providers noted significant time savings, estimating a 40%-50% reduction in documentation time.	“*[The scribe has] cut my actual documentation time down by probably 40-50% JUST by producing my HPI for me.*”
**Improved work-life balance (10 occurrences)**—The scribe helped providers complete documentation before leaving work, reducing the need for after-hours charting and improving personal time.	“*I was consistently taking home 3 or 4 patient notes per day, without being able to address my inbox at all. Now I leave work on Fridays with my inbox empty and all patient notes signed.*”
**Enhanced patient engagement (9 occurrences)**—Clinicians appreciated the ability to focus more on their patients rather than typing, leading to improved rapport and communication.	“*It makes the families feel like the physician is more engaged because we actually are. I have been holding more babies and coloring with more toddlers that ever before.*”
**AI Integration and formatting issues (7 occurrences)**—Some providers experienced challenges with how the scribe structured notes, including items in the wrong sections, difficulties capturing pertinent negatives, and challenges with template integration.	“*…even though I discuss specifics of pathophysiology with parents and patients (in lay language), the app only includes general discussions of our assessment and management plans and omits specifics.*”
**Challenges in behavioral health use (6 occurrences)**—Behavioral health providers reported that the scribe struggled to capture suicide risk assessments, mental status exams, and small progress indicators.	“*I cannot figure out how to do [a mental status exam]. I attempted to record this after the patient left, but the AI does not pick it up.*”
**Concerns over AI bias and consent (5 occurrences)**—Some patients and families declined to participate due to privacy concerns. Pediatricians also questioned whether parental consent was required for each visit or if one-time consent was sufficient.	“*Most patients and families have been happy to allow me to use it, though some have expressed concerns about AI and have declined.*” “*…if parents consent at the first session, do they need to continue to consent each session? Is there a certain age that the patient could consent without parent being there?*”

## Discussion

This is the first study to analyze digital scribe use among pediatric healthcare providers. We found significant reductions in documentation time, cognitive burden, and provider burnout, with neutral provider assessment of note quality. Time savings were similar to prior adult studies.^27^^,^^51^^,^^56–58^ Similarity with the adult literature is reassuring, as digital scribe performance may be meaningfully different in pediatric contexts. While EHR documentation time for pediatricians is similar to adult providers’, pediatric healthcare visits themselves are markedly different from adults’.^59^ Children are typically accompanied by an adult, requiring a digital scribe to differentiate between history relayed by the child and that of the caregiver. Furthermore, children experience both illness and relay medical history differently than adults depending on developmental stage, adding to summarization challenges for a digital scribe.

This study was also novel in demonstrating a significant reduction in provider burnout associated with digital scribe implementation, and in relating those reductions in burnout to decreased temporal and cognitive burden of documentation.^51^^,^^55–58^ While prior studies of human scribes have shown improvements in provider burnout, previous studies of digital scribes have failed to reliably demonstrate improvements in burnout despite differences in documentation time.^41–44^^,^^51^^,^^55–58^ In the present study, more than three-quarters of providers directly attributed improved job satisfaction to use of the digital scribe. These improvements are meaningful, as burnout is correlated with intentions to leave medicine among pediatricians.^83^ Interestingly, in thematic analysis, decreased cognitive burden occurred more often than decreased documentation time, with several comments suggesting that providers experienced cognitive distraction from incomplete documentation when trying to perform more clinically important tasks (such as operating). These comments may offer insight as to why there was a large reduction in provider burnout despite relatively modest time savings of only 1.5 hours per provider-week. Prior work has shown improvements in burnout when evaluating LLM drafts of patient message replies despite no significant time savings.^84^ Taken together, these data may suggest that reductions in cognitive load of EHR task are more important than absolute time savings when attempting to reduce provider burnout. Importantly, our study specifically did not require clinicians to increase patient volume in order to access a digital scribe, differing from some prior work.^61^ Whether this decision impacted clinician burnout is unclear, though physicians have previously cited increased productivity targets as a concern regarding digital scribe use.^55^ Furthermore, in the present study, there was no significant increase in the proportion of providers who felt able to add another patient to their clinic day after digital scribe adoption. Thus, “replacing” cognitive burden of EHR tasks with increased productivity requirements may also be counterproductive when attempting to reduce provider burnout.

In the present study, providers estimated 1.1 hours of time saved outside of work hours. EHR usage records suggested approximately 1.5 hours of total weekly time savings, but no significant savings outside of scheduled hours. This is similar to prior studies, where provider estimates of time outside of work are often higher than those calculated in EHR logs.^29^^,^^85^ Given this, others have expressed concern that EHR usage records may underestimate documentation burden due to excluding 30 minutes before and after the last scheduled appointment, ignoring work done between clinic appointments (eg, during lunch breaks or administrative time), and using a 5-second inactivity period to censor active session time.^86^^,^^87^ Providers may consider all of these periods to be “outside scheduled hours” even if not calculated as such by the EHR. Regardless of which method is more accurate, prior work correlating EHR use with burnout has used self-reported time.^15^^,^^17^

In the case of the present study, reductions in provider burnout prompted purchase of an enterprise license of the digital scribe, and rollout is ongoing across the organization. Our system is now beginning phased implementation across multiple outpatient clinics. Additionally, we plan to study the use of the tool in both emergency department and inpatient settings during the upcoming rollout. Further study is necessary to determine whether time savings and reductions in cognitive load and burnout are generalizable to these settings. While medical documentation is ubiquitous across specialties, EHR documentation time and workflows (eg, whether notes are completed during or after an encounter) varies widely depending on clinical context and provider training.^88–90^ Additionally, demographic differences in subspecialty composition may influence documentation time.^91^

This study has important limitations. First, all pilot users were volunteers; benefits seen in volunteer samples are often less impressive when applied across an entire organization. Further studies are needed to determine whether the effect is maintained as the health system deploys the digital scribe broadly. Second, scribe use in the present pilot study was only approximately 6 months in duration. Long-term analysis is needed to determine if effects are sustained. Third, this study did not assess cost-effectiveness. Digital scribes cost several thousand dollars per year, though improved physician productivity and retention related to decreased burnout is valued at hundreds of thousands of dollars per year.^92^ Fourth, long-term studies are needed to determine if benefits are sustained beyond a pilot period. Fifth, caregiver satisfaction surveys were collected from a random sampling of clinical encounters per routine clinical practice, so may be subject to random variation that may underestimate any effect of digital scribe implementation. Sixth, during the pre-scribe period, some clinical departments underwent note template revisions to improve documentation efficiency. This may reduce the measured effect of the digital scribe on documentation time in pretest-posttest analysis and bias towards the null.

## Conclusion

Digital scribe use was associated with significant improvements in documentation time, cognitive burden, and burnout among pediatric healthcare providers in this pilot study. More work is needed to determine whether these findings will be representative of ongoing organization-wide implementation.

## Data Availability

The data underlying this article were provided by Epic (Verona, WI) and Abridge (Philadelphia, PA) under license/by permission. Data will be shared on request to the corresponding author with permission of Epic and Abridge.
